# Supplementary material for: Reassessment of miRNA variant (isomiRs) composition by small RNA sequencing
Source: Cell Rep Methods. 2023 May 16;3(5):100480. doi: 10.1016/j.crmeth.2023.100480 (PMC10261927; doi:10.1016/j.crmeth.2023.100480)
Supplement: Document S1. Figures S1 and S2 and Table S1 [file mmc1.pdf]

Cell Reports Methods, Volume *3*

Supplemental information

Reassessment of miRNA variant (isomiRs) composition by small RNA sequencing

Cristina Gómez-Martín, Ernesto Aparicio-Puerta, Monique A.J. van Eijndhoven, José M. Medina, Michael Hackenberg, and D. Michiel Pegtel

Supplemental Information

Classification	Sequence
Canonical mature miRNA	CAACACCAGUCGAUGGGCUGU
NucVar: miRNA with internal sequence variation	CAACACCAGUCGUUGGGCUGU
NTA: Non Templated Additions	CAACACCAGUCGAUGGGCUGUA
	CAACACCAGUCGAUGGGCUGUU
	CAACACCAGUCGAUGGGCUGUCC
	CAACACCAGUCGAUGGGCUGUG
Length variants	
lv3p: Lenght variants 3′ end	CAACACCAGUCGAUGGGCU--
	CAACACCAGTCGTTGGGCUGUCT
lv5p: Length variants 5′ end	--ACACCAGUCGAUGGGCUGU
	GGCAACACCAGUCGAUGGGCUGU
mv: multiple length variants	GCAACACCAGUCGAUGGGCUG-

Supplementary Figure 1: IsomiR classification, Related to Figure 1.

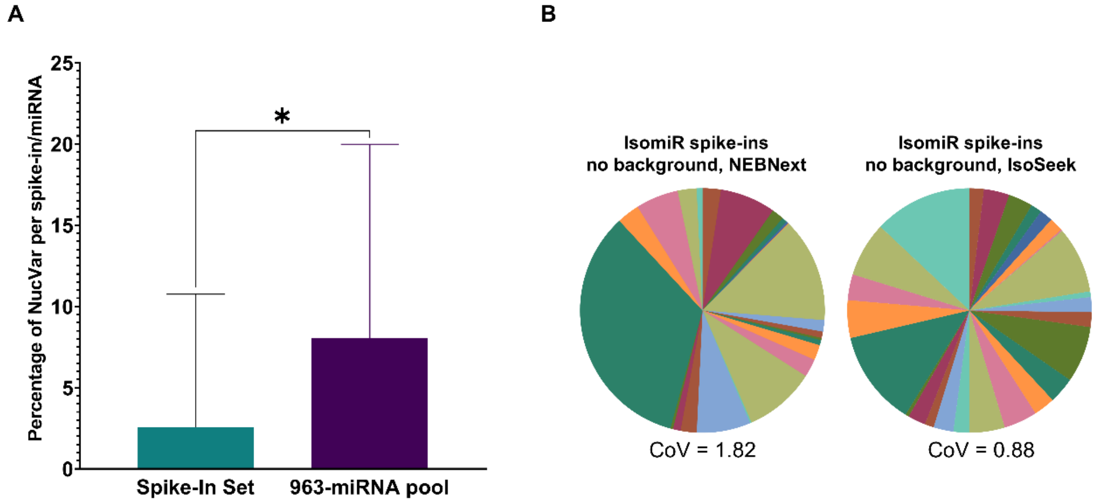

Supplementary Figure 2. Comparison of NucVar isomiRs in the 936-miRNA pool and custom isomiR spike.in set, Related to Figure 2. A) Percentage of NucVar isomiRs per Spike-in/miRNA in the 26 artificial spike-in set (n=3) and the 963-miRNA pool (n=3), both sequenced using Isoseek. The height of the bar represents the mean and the error bar the SD. **B)** Distribution of 26 isomiR spike-ins without biological background. Library preparation is done using NEBNext (left) and IsoSeek (5N) (right).

	TruSeq	CleanTag	NEBNext	AQ-RNA	NEXTflex	QIAseq	IsoSeek	4N-X	4N-G	AQseq
**Randomized-end adapters**	×	×	×	✓	✓	×	✓	✓	✓	✓
**Unique Molecular Identifiers**	×	×	×	×	×	12N	5N x 2	×	×	×
**Hybridation of RT primer**	After both adapters ligation	After both adapters ligation	Between 3' and 5' adapter ligation	Between 3' and 5' adapter ligation	After both adapters ligation	After both adapters ligation	Between 3' and 5' adapter ligation	After both adapters ligation	After both adapters ligation	After both adapters ligation
**Other**		Optimized structure modifications at the termini of the adapters to avoid adapter dimers					20% PEG at adapter ligation			20% PEG at adapter ligation

Supplementary Table 1. Schematic overview of the different sequencing protocol characteristics, Related to Figure 1.
