# Supplementary material for: Reassessment of miRNA variant (isomiRs) composition by small RNA sequencing
Source: Cell Rep Methods. 2023 May 16;3(5):100480. doi: 10.1016/j.crmeth.2023.100480 (PMC10261927; doi:10.1016/j.crmeth.2023.100480)
Supplement: Document S2. Article plus supplemental information [file mmc2.pdf]

Cell Reports Methods

Resource

Reassessment of miRNA variant (isomiRs) composition by small RNA sequencing

Graphical abstract

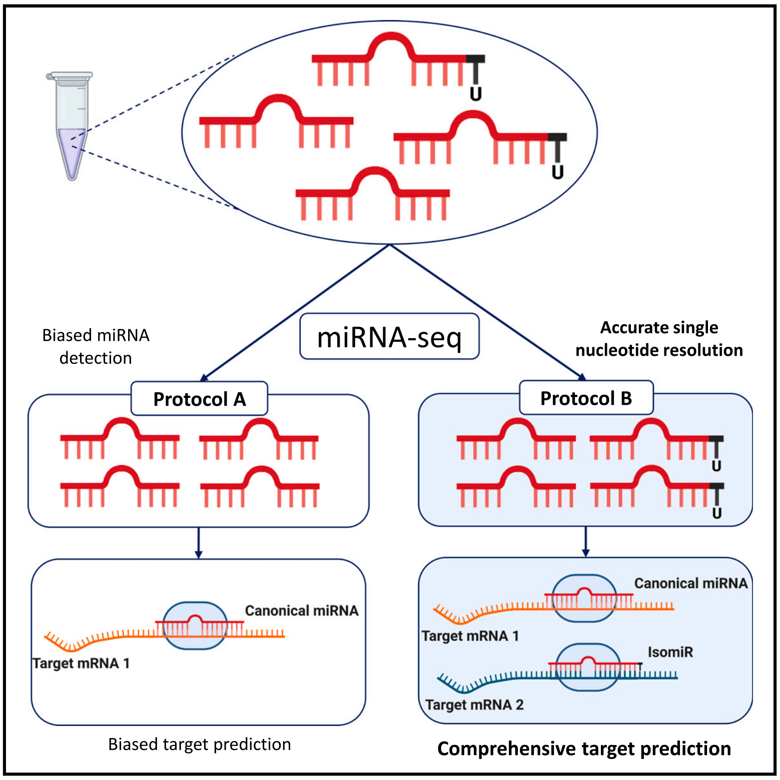

Authors

Cristina Gómez-Martín, Ernesto Aparicio-Puerta, Monique A.J. van Eijndhoven, José M. Medina, Michael Hackenberg, D. Michiel Pegtel

Correspondence

hackenberg@go.ugr.es (M.H.),
d.pegtel@amsterdamumc.nl (D.M.P.)

In brief

Gómez-Martín et al. demonstrate that isomiRs are non-artifactual variants of miRNAs and that most small RNA sequencing protocols, especially those with randomized adapters, detect them with high accuracy. Single-nucleotide accuracy is crucial for making predictions about biological function. The work provides a reference for researchers interested in small ncRNA.

Highlights

- IsomiRs are not a consequence of library artifacts in most RNA-seq protocols
- Randomized adapter protocols outperform fixed adapters in isomiR calling accuracy
- NTA-U calling is dependent on high-single-nucleotide-resolution performance
- NTA-U isomiR calling affects mRNA target prediction

Gómez-Martín et al., 2023, Cell Reports Methods *3*, 100480
May 22, 2023 © 2023 The Author(s).
https://doi.org/10.1016/j.crmeth.2023.100480

CellPress

Cell Reports Methods

CellPress
OPEN ACCESS

Resource

Reassessment of miRNA variant (isomiRs) composition by small RNA sequencing

Cristina Gómez-Martín,[1,2] Ernesto Aparicio-Puerta,[3,8] Monique A.J. van Eijndhoven,[1,2,8] José M. Medina,[4,5] Michael Hackenberg,[4,5,6,7,*] and D. Michiel Pegtel[1,2,9,*]
[1]Amsterdam UMC Location Vrije Universiteit Amsterdam, Pathology, De Boelelaan, 1117 Amsterdam, the Netherlands
[2]Cancer Center Amsterdam, Imaging and Biomarkers, Amsterdam, the Netherlands
[3]Chair for Clinical Bioinformatics, Saarland University, 66123 Saarbrücken, Germany
[4]Department of Genetics, Faculty of Science, University of Granada, 18071 Granada, Spain
[5]Bioinformatics Laboratory, Biotechnology Institute, Centro de Investigación Biomédica, PTS, Avda. del Conocimiento s/n, 18100 Granada, Spain
[6]Instituto de Investigación Biosanitaria ibs.GRANADA, University of Granada, 18071 Granada, Spain
[7]Excellence Research Unit "Modelling Nature" (MNat), University of Granada, 18071 Granada, Spain
[8]These authors contributed equally
[9]Lead contact
*Correspondence: hackenberg@go.ugr.es (M.H.), d.pegtel@amsterdamumc.nl (D.M.P.)
https://doi.org/10.1016/j.crmeth.2023.100480

MOTIVATION Sequence variants (isomiRs) of mature microRNAs can be detected by high-throughput sequencing methods. However, because isomiRs differ from the canonical (mature) miRNA sequence often by only a single nucleotide, most protocols introduce errors that may negatively influence biological interpretation. To investigate how sequencing protocols impact isomiR calling, we conducted a comprehensive comparison of 10 small RNA-seq protocols, providing a comprehensive reference guide for miRNA/isomiR interpretation by sequencing.

SUMMARY

IsomiRs, sequence variants of mature microRNAs, are usually detected and quantified using high-throughput sequencing. Many examples of their biological relevance have been reported, but sequencing artifacts identified as artificial variants might bias biological inference and therefore need to be ideally avoided. We conducted a comprehensive evaluation of 10 different small RNA sequencing protocols, exploring both a theoretically isomiR-free pool of synthetic miRNAs and HEK293T cells. We calculated that, with the exception of two protocols, less than 5% of miRNA reads can be attributed to library preparation artifacts. Randomized-end adapter protocols showed superior accuracy, with 40% of true biological isomiRs. Nevertheless, we demonstrate concordance across protocols for selected miRNAs in non-templated uridyl additions. Notably, NTA-U calling and isomiR target prediction can be inaccurate when using protocols with poor single-nucleotide resolution. Our results highlight the relevance of protocol choice for biological isomiRs detection and annotation, which has key potential implications for biomedical applications.

INTRODUCTION

MicroRNAs (miRNAs) are small non-coding RNA transcripts widely studied for their role in gene expression regulation, which they exert by imperfectly pairing to a target messenger RNA (mRNA).[1] Expression profiles of the 2,600 reported human miRNAs over different tissues and pathophysiological conditions are a first step to understand the underlying biology and are useful for diagnostics[2] and therapeutics.[3] To generate comprehensive miRNA expression profiles, specific next-generation sequencing (NGS) protocols were developed. Initially, protocols were based on fixed (invariant) sequence adapters that introduced bias due to preferential ligation affinity to certain sequences, resulting in a high variability of less informative ncRNAs (Y-RNA, tRNA fragments).[4,5] To overcome this, recent sequencing protocols make use of random nucleotides at one or both sequencing adapters, thereby reducing detection bias of canonical miRNAs.[5–8] However, accurate detection of post-transcriptional modifications remains challenging, in particular for low-input applications such as extracellular miRNA profiling used for minimally invasive diagnostics.

Early NGS studies revealed that a significant proportion of miRNA reads in biological samples deviate in length and sequence from the 20–21 nucleotide canonical miRNAs.[9]

This is an open access article under the CC BY license (http://creativecommons.org/licenses/by/4.0/).

Among these variants, termed "isomiRs,"[10] one type is generated by post-transcriptional non-templated nucleotide additions (NTAs) by terminal nucleotidyl transferases.[11] These enzymes add up to 3 nucleotides to the 3′ end, leading to slightly elongated mature miRNAs that will thus deviate from the genomic sequence. Functional implications of such post-transcriptional modifications are altered miRNA stability[12] and/or different mRNA targets compared with the canonical sequence.[13] A recent study describes that dysfunctional isomiRs accumulate in cancer cells, and that enzymatically restoring their canonical function may have therapeutic value.[14] Indeed, modified miRNAs act as independent functional molecules that may support or counteract canonical miRNAs with diagnostic and prognostic implications. For instance, the targetome of one miR-411 isomiR that is upregulated in chronically ischemic human blood vessels has a very small overlap with the canonical miR-411 targetome.[15]

Due to their functional relevance, accurate discrimination between true isomiRs and sequencing or alignment artifacts is crucial to avoid misinterpretation of their biological function and diagnostic significance.[16] Therefore we analyzed and compared the presence of isomiRs in a large body of sequencing data generated by independent laboratories using 10 different protocols to evaluate protocol differences in detecting true biological isomiRs. To assess comparable samples, we selected sequencing datasets from the miRXplore Universal Reference, a pool consisting of 963 different chemically synthesized miRNAs (thus theoretically free of isomiRs) and libraries obtained from the HEK293T cell line. To avoid any bioinformatics processing bias, all sample data were uniformly processed using sRNAbench,[17] a broadly used miRNA analysis software that can also accommodate virtually any sequencing protocol. We also included samples from "IsoSeek," our thoroughly optimized[7] in-house 5N protocol that aims to account for all technical bias by using 5′ and 3′ randomized adapters, each of them with 5 random nucleotide unique molecular identifiers (UMIs) (5N) based on commercial adapters.[18] Our results show that regardless of the adapter strategy, the majority of protocols generate only minor library preparation artifacts and detect biological variants with relevant overlap, mostly 3′ end NTAs. Moreover, 4N and 5N protocols appear to be highly suitable for isomiR profiling of biological samples, whereas some protocols suffer from biased quantification and/or generation of artificial isomiRs.

RESULTS

Analysis of the 963-miRNA miRXplore reference pool reveals that the majority of small RNA-seq protocols generate low levels of false isomiRs

A recent study cautioned that small RNA-seq protocols generate a large proportion of non-biological miRNA variants that should be considered as artifacts.[8] We first scrutinized this claim by analyzing the canonical miRNA and isomiR profiles from 10 different protocols generated furthermore by different laboratories. Specifically, we analyzed data from a synthetic 963-miRNA reference pool (miRXplore Universal, Miltenyi Biotec) and HEK293T cellular RNA using commercial and non-commercial randomized-end adapter protocols (AQ-seq,[6] 4N-G and 4N-X,[5,6] NEXTflex, and our own 5N "IsoSeek" method[7]), one custom 2N adapter protocol (AQRNA-seq[8]), and three commercial invariant (fixed) adapter protocols (NEBNext,[13,19] TruSeq,[20] QIAseq,[21,22] and Clean-Tag[22]). The main characteristics of the different protocols are summarized in Table S1.

Mapping statistics of the 963-miRNA pool revealed that 7 out of the 9 protocols showed 75%–80% recovery of exact matches, i.e., canonical sequences (Figure 1A). AQRNA-seq and 4N-G protocols have a consistently lower percentage of canonical sequences but, in contrast, a higher percentage of 5′ end length variants (lv5p, see isomiR classification used in Figure S1) than the other protocols. NTA isomiRs (see Figure S1) should theoretically be absent in the reference pool as these samples were never in contact with nucleotidyl transferases. Indeed, this subclass is almost not detected by any protocol (~1% for each NTA class), with the exception of NEBNext, which appears to generate the most artifacts (see also Table 1A). However, more prominent were internal single mismatches (NucVar) that were present in ~10%–12% of the miRNA-mapped reads in all protocols, except for 2 samples from the 4N-G protocol with only ~4% of NucVar. This could be explained by a massive number of extensively trimmed 5′ length variants that drastically reduce the percentage of canonical sequences (Figure 1A and Table 1A). In fact, all other protocols have only ~2% length variants (Figure 1A and Table 1A), often only lacking a single nucleotide at 3′ or rarely at the 5′ end (Table 1A).

In contrast to the reference pool, the analysis of the isomiR distribution of the 23 HEK293T libraries revealed a different pattern. All protocols showed that ~40%–50% of the miRNA-mapped reads represented the exact mature miRNA sequence (canonical) as annotated in miRBase (Figure 1B). The most prominent isomiR class is the 3′ end length variants (lv3p), which are presumably generated by "sloppy" Drosha or Dicer cleavage or the action of exonucleases.[1] Finally, approximately 10%–20% of the miRNA reads belong to NTA isomiRs, similarly to previously found in other studies.[11]

It was unexpected that the percentage of NucVar isomiRs was 3- to 4-fold smaller in cells (~4%) compared with the reference pool (~12%). This could indicate that, rather than artifacts of library preparation, artificial NucVar isomiRs represent errors already introduced during oligosynthesis of the 963-miRNAs pool.[23] To investigate this possibility, we measured the presence of artificial NucVar isomiRs in a customized panel of 26 synthetic isomiRs, and we then compared the resulting percentage to the 963-miRNA pool results (Figure S2A). Interestingly, we found a significantly lower percentage of NucVar in our customized spike-in set. Taking into account that our spike-in set was generated with the highest quality control standards, this suggests the presence of oligosynthesis errors in the commercial 963-miRNA pool.

Moreover, we directly compared the performance of IsoSeek with the fixed adapter NEBNext protocol using the same 26 synthetic isomiRs that were sequenced without any biological background (Figure S2B). The distribution of the different isomiRs was considerably more equal in the IsoSeek samples, with 0.88 coefficient of variation (CoV), compared with NEBNext with 1.82 CoV, indicating a major reduction in bias.

CellPress
OPEN ACCESS

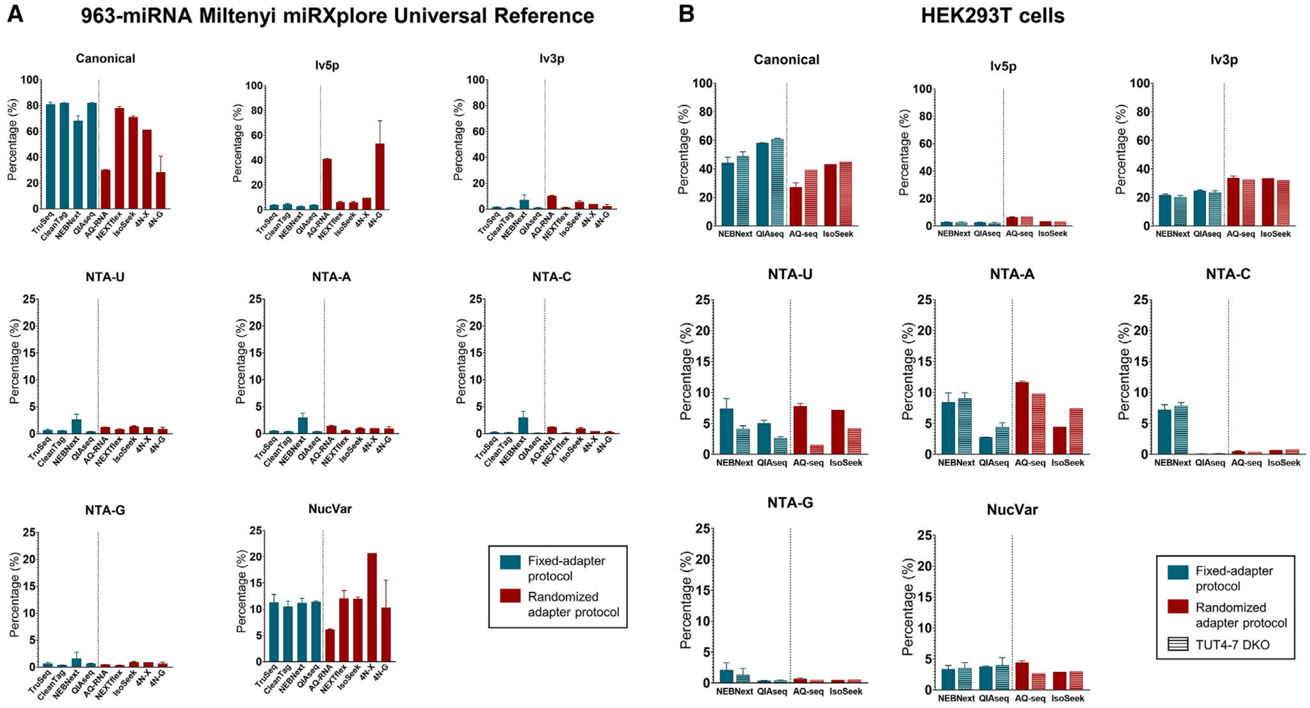

Figure 1. IsomiR distribution in 963-miRNA Mintenyi miRXplore Universal Reference pool libraries and HEK293T cells

(A) IsomiR distribution in 62 libraries from the 963-miRNA Miltenyi miRXplore Universal Reference pool. A high percentage of canonical miRNAs (~75%) is detected with almost all protocols, with the exception of 4N Giraldez in-house protocol (4N-G) and AQRNA-seq protocol that have a high percentage of 5′ end length variants and therefore a low percentage of canonical isoforms. The pool also contains a relatively large (~10%) of NucVar isomiRs (only one mismatch compared to the canonical sequence), which was detected in all analyzed libraries. The height of each bar represents the mean percentage and the error bars the SD.

(B) IsomiR distribution in 23 libraries from HEK293T cells. All small RNA sequencing protocols show ~40% of canonical miRNAs. The most abundant isomiRs are Iv3p (~25%) and NTA-U/A (~10% each one) and also NTA-C only in NEBNext samples (~10%). The bars with a striped pattern indicate HEK293T cells with a TUT4/7 DKO. In all the protocols, a reduction of the NTA-U level in the DKO cells is observed. The height of each bar represents the mean percentage and the error bars the SD.

Analysis of length variant isomiRs in the 963-miRNA set and HEK293T cells reveals 3′ end length variants outnumber 5′ end length variants

We next performed an in-depth analysis of the four different length variant classes, i.e., extended or trimmed at the 5′ or 3′ end (Figure S1). As can be observed in Table 1A, the majority of the protocols showed that samples from the 963-miRNA pool have a relatively low percentage of length variants (~5%). This contrasts with the HEK293T cells (Table 1B) where the percentage of length variants is much (6-fold) higher (~30%), suggesting a biological origin. In fact, in the case of the 963-miRNA pool samples, the most prominent length variants are at the 5′ end, while the HEK293T cells had a high presence of lv3p isomiRs, which are consistent with "sloppy" Drosha or Dicer cleavage or the action of exonucleases.[12] Therefore, most 5′ truncated miRNAs (lv5pT) and other 5′ length variants are very likely due to library preparation artifacts.

A high proportion of the NucVar in HEK293T cell isomiRs point to biological origin

We next looked in more detail into the different classes of single-nucleotide changes (NucVar, see Figure S1) in HEK293T cells. A high percentage of T>C and A>G single-nucleotide changes (in red) were consistently detected among the 4 protocols (NEBNext, QIAseq, AQ-seq, and IsoSeek) compared to all the other potential changes, which showed a much lower percentage (Figure 2A).

We then compared the percentage of these two NucVar types, T>C (Figure 2B) and A>G (Figure 2C), in HEK293T cells to the percentage in the 963-miRNA pool samples sequenced with the same protocol. The percentage of both variants in the HEK293T cells was significantly higher in both cases (p value < 0.01), regardless of the sequencing protocol used. Interestingly, these two variants specifically may be caused by enzymatic activity. Specifically, the A>G conversion can be a result of the ADAR enzyme activity[24,25] and, in the case of T>C, a result of APOBEC3C action.[26]

4 different small RNA protocols robustly detect NTA-U/A isomiRs in HEK293T cells that are absent in the artificial reference pool

We observed that the percentage of NTA isomiRs in the 963-miRNA pool was consistently lower (~1% per NTA class) than the percentage in HEK293T cells (~20% total NTA), as

Table 1. Percentage of non-templated addition (NTAs) isomiRs in 62 libraries from the 963-miRNA Miltenyi miRXplore Universal Reference pool and 12 libraries from HEK293T cells

	963-miRNA Miltenyi miRXplore Universal Reference			
A	NTA-U	NTA-A	NTA-C	NTA-G
TruSeq	0.67	0.53	0.26	0.69
CleanTag	0.61	0.39	0.24	0.39
NEBNext	2.63	2.97	2.99	1.62
AQRNA	1.25	1.43	1.27	0.5
QIAseq	0.41	0.39	0.15	0.67
NextFlex	0.81	0.6	0.19	0.34
IsoSeek	1.41	1.01	0.96	0.96
4N-X	1.19	1.08	0.95	0.44
4N-G	0.85	0.96	0.32	0.67

	HEK293T Cells			
B	NTA-U	NTA-A	NTA-C	NTA-G
NEBNext	7.4	8.41	2.13	7.21
QIAseq	5.03	2.75	0.1	0.35
AQ-seq	7.48	11.16	0.46	0.67
IsoSeek	7.16	4.45	0.68	0.51

A very low percentage (~1%) of all NTAs is observed in the pool, with the highest value from the samples of the NEBNext protocol (~3%). In contrast, a high percentage of NTA-U/A is observed in the HEK293T (~15% with the exception of QIAseq protocol), while NTA-C/G (with no biological function underlined) percentages remain low. Only one fixed adapter protocol (NEBNext) showed a more significant higher percentage of NTA-C/G isomiRs.

shown in Figure 1. We then looked deeply into the different NTA isomiRs, i.e., NTA-A, NTA-U, NTA-C, and NTA-G (Table 2). In HEK293T cells, approximately 10%–30% of the total miRNA reads belonged to NTA isomiRs, similar to previous studies,[11] which is up to 20-fold higher than in the reference pool libraries. The majority are NTA-U and NTA-A isomiRs, while NTA-C and NTA-G are hardly detected using AQ-seq and IsoSeek (Figure 1B and Table 2B). Moreover, in addition to a lower NTA percentage detected, a more equal and possibly random distribution of the percentage of the different NTA classes was observed (Table 2A). Overall, randomized adapter protocols reveal the more equal percentage of NTA distribution and the lowest percentage of artificial NTA in the 963-miRNA pool samples in agreement with prior observations.[11] Despite minor differences, we conclude that the majority of NTA-A and NTA-U classes that are detected in HEK293T cells represent true biological isomiRs.

TUT4/7 knockout HEK293 cells reveal concordance and differences between protocols in the detection of uridylated isomiRs (NTA-U)

To assign specificity of the protocols for biological isomiR detection, we studied the biological activity of nucleotidyl (uridyl) transferases TUT4 and TUT7 in HEK293T CRISPR-Cas-mediated double knockout (DKO) HEK293T cells. TUT4 and TUT7 enzymes synthetize the addition of uridine nucleotides at the 3′ end of miRNAs.[11] To fully establish that small RNA sequencing data obtained by various protocols contain biologically generated isomiRs, and because uridylation may be cell type specific,[27] we analyzed 11 libraries from TUT4/7 DKO HEK293T cells[13] prepared with 4 different protocols in 6 different laboratories (see striped bars in Figure 1B). In agreement with knockdown studies using a NanoString-based detection in a different cell type,[11] all small RNA sequencing protocols showed reduced percentages of NTA-U in the TUT4/7 DKO cell lines compared with wild-type (WT) counterparts (Figure 1B).

To establish the reproducibility of NTA-U isomiR detection by small RNA sequencing protocols, we assessed the detection of miRNAs with an uridylation ratio drop of at least 2-fold in TUT4/7 DKO HEK293T cells compared to the parental cells. We used data from 6 independent studies that employed 4 different protocols (NEBNext, QIAseq, AQ-seq, and IsoSeek). We identified 20 miRNAs (6% of all differentially uridylated miRNAs by at least 1 of the protocols) that were consistently detected as differentially uridylated due to the lack of TUT4/7 enzymes in all studies (Figure 3). We validated this overlap by comparison to a random selection from the total list of differentially uridylated miRNAs (see more details in STAR Methods), yielding a highly significant Z score of 28.5. These analyses indicate that the observed differences in NTA-Us between WT and DKO cell lines with multiple protocols are not random but are driven by the depletion of TUT4/7 enzymes and thus biologically motivated.

Differences across protocols in detection of TUT4/7-dependent miRNA uridylation

In order to estimate potential biases in the detection of NTA isomiRs, we performed a deep analysis into the NTA class distribution in parental and TUT4/7 DKO HEK293T cells. Using AQ-seq, IsoSeek, and QIAseq, we observed an overall reduction of NTA-U of 25%, 24%, and 34% respectively in TUT4/7 DKO cells as compared to parental cells (Figure 4A). However, with the fixed adapter protocol NEBNext, this reduction was only 4% (Figure 4A). Interestingly, using randomized adapter protocols, AQ-seq and IsoSeek, the global NTA-U reduction observed in DKO cells (~24%) was complemented by a similar increase of the NTA-A fraction (~23%). This phenomenon was recently described in cancer cells,[27] and this subtle but significant correlation was not seen in the fixed adapter protocols (Figure 4A). On the other hand, the QIAseq protocol detected a significant decrease on uridylation but not a clear increase in NTA-A isomiRs. Instead, NTA-C isomiRs were increased. Taking into account that such isomiRs have not been described as enzymatically driven, we cannot rule out the possibility of technical artifacts presence.

We next investigated those miRNAs that displayed a clear difference in uridylation percentage in the DKO compared with the WT cells based on IsoSeek protocol. In most of the miRNAs where 3′ end uridylation was reduced in the DKO, we observed an increase in 3′ end adenylation (Figure 4B). However, this was not the case for guadinylation and cytidylation (Figures 4C and 4D respectively). Taken together, these results suggest that a specific increase in adenylation of selected miRNAs in DKO cells occurs in the absence of urydilation. These

CellPress
OPEN ACCESS

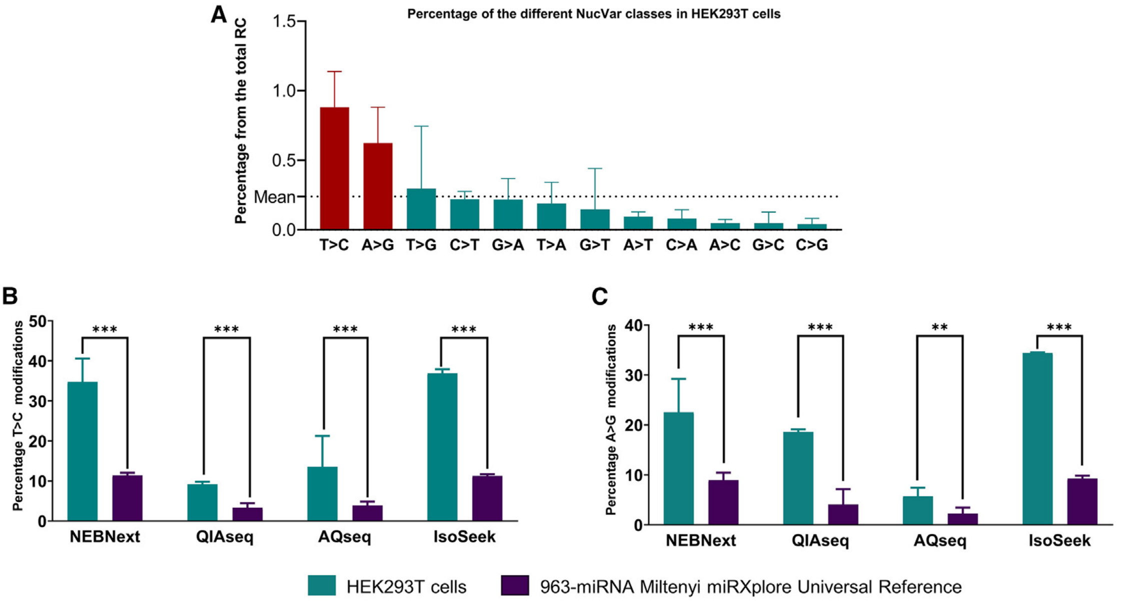

Figure 2. NucVar isomiRs profiling in HEK293T cells compared to the 963-miRNA pool

(A) Percentage of HEK293T different NucVar isomiRs. The most prominent classes were T>C and A>G changes. The height of each bar represents the mean percentage of each NucVar class of the total miRNA read counts of 4 different protocols (IsoSeek, QIAseq, AQ-seq, NEBNext, n = 20), and the error bar represents the SD. The mean line represents the mean percentage of all NucVar classes in all protocols.

(B and C) Percentage of T>C and A>G modifications respectively compared to the total number of reads mapping to NucVar in both HEK293T cells and the 963-miRNA pool samples. In all the comparisons, the percentage of the variants in the cells was statistically significantly higher than in the 963-miRNA pool. The height of the bars represents the mean percentage and the error bars the SD.

observations indicate that 3′ end NTA modification is a non-random biological process caused by competing NTA (uridylase vs. adynelase) nuclease activities. We also evaluated the uridylation of 3p and 5p arm-derived miRNAs and observed that TUT4/7-dependent uridylation occurs predominantly on 3p miRNAs. This preference is especially pronounced when using randomized adapter protocols with reduced bias while being much less evident with the NEBNext fixed adapter protocol (Figure 4E).

Biased detection of miRNA uridylation can affect target prediction

To investigate whether differences in isomiR detection between protocols influence target prediction strategies,[13] we compared the top 10 most uridylated miRNAs identified per protocol (Figure 5A). miRNA hsa-miR-30e-3p, which has a role in cancer,[28,29] is heavily mono-uridylated according to randomized adapter protocols, while with NEBNext, the exact mature sequence is more abundant (Figure 5B). Importantly, only IsoSeek and QIAseq protocols show a clear decrease in uridylation in TUT4/7 DKO cells and an increase in adenylation (Figure 5B).

Having identified a biological isomiR, we performed target prediction for both the canonical hsa-miR-30e-3p and the 3′ end mono-uridylated isoform. We observed many unique potential target genes of both the canonical miR-30e-3p and the mono-uridylated isomiR. This is in agreement with the recently described tail-U-mediated repression (TUMR) as described by Yang et al.[13] In fact, we found many more isomiR unique targets than overlapping targets (Figure 5C). These observations may have critical implications for the biological function of these closely related isomiRs with a highly divergent targetome. Indeed, pathway enrichment analysis of the canonical targetome (Figure 5D) and the TUMR targetome (Figure 5E) showed that different pathways are enriched, leading to physiological changes. This analysis exemplifies the potential consequences of inaccurate isomiR detection derived from suboptimal choice of a sequencing protocol.

DISCUSSION

The advent of NGS has revealed that miRNAs, apart from the 21/22 nucleotide canonical (mature) genomic miRNA sequences, are comprised of many variants known as isomiRs. We show here that distinguishing true isomiRs from technical artifacts and sequencing errors is of high importance, as it may result in misinterpretation of their biological function (i.e., targetome),[13] and diagnostic[16] and potential therapeutic significance.[14] To demonstrate this, we thoroughly compared sequencing data from HEK293T cells and a 963-synthetic miRNA pool that should, theoretically, be free of any isomiRs. 95 samples were analyzed and sequenced with in total 10 different protocols including widely used fixed (or invariant) commercial adapters and newer protocols that make use of randomized adapters and other customized library preparation procedures.

We report several novel findings, some of special relevance when studying the biology of isomiRs. First, when benchmarking protocols using a standard reference set, it is of key importance that the quality of all oligos (reference sets, primers,

Table 2. Mean percentage of the different length variants isomiRs in 62 libraries from the 963-miRNA Miltenyi miRXplore Universal Reference pool and 12 libraries from HEK293T cells

A	963-miRNA Miltenyi miRXplore Universal Reference			
	lv5pT	lv5pE	lv3pT	lv3pE
TruSeq	3.77	0.03	1.31	0.22
CleanTag	4.55	0.05	0.93	0.28
NEBNext	2.67	0.03	7.08	0.13
AQRNA	26.25	3.47	9.71	0.37
QIAseq	3.74	0.05	0.98	0.28
NextFlex	6.11	0.08	1.02	0.32
IsoSeek	5.89	0.08	5.09	0.69
4N-X	9.64	0.24	3.77	0.44
4N-G	52.77	0.15	2.21	0.24

B	HEK293T Cells			
	lv5pT	lv5pE	lv3pT	lv3pE
NEBNext	2.14	0.53	9.86	11.9
QIAseq	3	0.19	15.04	11.77
AQ-seq	4.86	1.23	16.6	18.01
IsoSeek	0.59	2.93	19.89	13.49

A low percentage (~10%) of length variants is observed in the pool in all protocols except the 4N in-house protocol from Giraldez et al.[5] (4N-G) and AQRNA-seq protocol. Both these protocols seem to create a high percentage of 5′ trimmed length variants. In contrast, the length variants observed in the HEK293T cells are mainly at the 3′ end. The color intensity of each cell is relative to its value.

and adapters) is extremely high, as synthesis errors negatively influence the results (Figures 1 and S2 and Tables 1 and 2). Second, some protocols may generate high numbers of library preparation artifacts (Figure 1 and Tables 1 and 2). Third, randomization of adapter sequences at the 3′ and 5′ ends strongly reduces ligation bias. Fourth, UMI-correction can reduce amplification bias but appears less essential when RNA input is not limiting. Fifth, to determine the biological relevance of isomiRs, analysis of control cells that lack relevant NTA-modifying enzymes is advised. Sixth, we determined higher percentages of T>C and A>G NucVar changes in cells when compared with the reference pool. This is in line with a biological role as these modifications are the only mutations known caused by RNA-editing enzymes.[24–26] Seventh, uridylation on 3p-arm miRNAs is much more frequent than 5p miRNAs (Figure 4E), confirming the idea that uridylation generally occurs after Drosha cleavage but before Dicer processing.[30] Finally, only randomized adapter protocols detect NTA miRNA substrate competition (Figure 4), a recently discovered biological phenomenon in cancer cells.[27]

What is the current experimental evidence for a biological role of isomiRs? In cartilage, a miR-140-3p isomiR is the dominant and active form, with a completely different seed and targetome.[31] In addition, there is evidence that shRNA-induced liver toxicity is solely related to competition with a major isoform of miR-122.[32] Moreover, some classes of isomiRs are preferentially sorted into communicating extracellular vesicles,[33,34] which could have important biological and diagnostic implications. Most strikingly, Qi et al. recently reported that expression of a plant nucleotidyl-transferase (RDR1) in human cancer cell lines blocks proliferation by targeting cell cycle genes and that these effects were dependent on the miRNA pathway.[14] It appeared that miRNA duplex isoforms with 1-nt-shorter 3′ ends cannot be efficiently loaded onto the Argonaute complex and accumulate in human tumors. RDR1 expression restored the regular 2-nt overhang structure of miRNA duplexes, thereby rescuing the defective miRNA pathway in mouse xenograft models, which suppressed tumor growth.[14] In another study, an isomiR sequence of miR-411 was 5-fold more abundant than the canonical form in primary human vascular cells and in venous tissue samples from patients with peripheral artery disease. The authors show that isomiR-411 and canonical miR-411 expression is differentially regulated under ischemic conditions in a murine hindlimb ischemia model.[15] Finally, isomiRs may act as cancer biomarkers and function as either allies or antagonists of their canonical counterparts.[35] By using cell, and not tissue, data, McCall et al. observed many cell-specific differences in the isomiR composition of miRNAs.[36] Notably of 205 common miRNAs, the most abundant sequence did not match the reported sequence in mirBase.org.[36]

Since a large body of studies shows that isomiRs have a biological function, it is thus critical to choose reliable library protocols and mapping strategies. We observed that 4-5N randomized adapters approaches resulted in the most unbiased protocols, showing the lowest amount of NTA percentage in the 963-miRNA reference pool samples. This is in agreement with previous observations only considering the exact miRNA sequences.[11] IsoSeek and AQ-seq detect profound global changes in the miRNA uridylome in HEK293T cells, while this was not observed using the fixed adapter NEBNext protocol. Nevertheless, all protocols determined that hsa-miR-760, which has been correlated with breast cancer progression,[37] is a heavily uridylated miRNA (Figure 5A). Importantly, exploiting CRISPR-Cas9-generated TUT4/7 DKO cells, we could identify miRNAs that are specifically uridylated by the combined action of these TuTases. We identified with multiple protocols that at least 20 miRNAs (6%) are selectively uridylated by these enzymes (Figure 3), a biologically relevant observation that was also highly significant (*Z* score = 28.5). NTAs by TUT4 and TUT7 triggers "arm-switching," changing the repressive activity of modified miRNAs.[30] The TUMR is abolished in cells lacking the uridylation enzymes TUT4 and TUT7.[13] We show that the canonical and mono-uridylated miR-30e-3p targetome differ dramatically (Figure 5), which highlights that the correct interpretation of this biological phenomenon relies on appropriate protocol selection.

In summary, this study unveils the importance of an appropriate sequencing method for isomiR analysis. Randomized adapter-based protocols outperformed those with fixed adapters on this end. This is of crucial importance for the detection of non-templated additions (especially NTA-Us and NTA-As), which are miRNA modifications of high biological relevance that are underestimated or overestimated by certain protocols due to their different biases. Accurate

CellPress
OPEN ACCESS

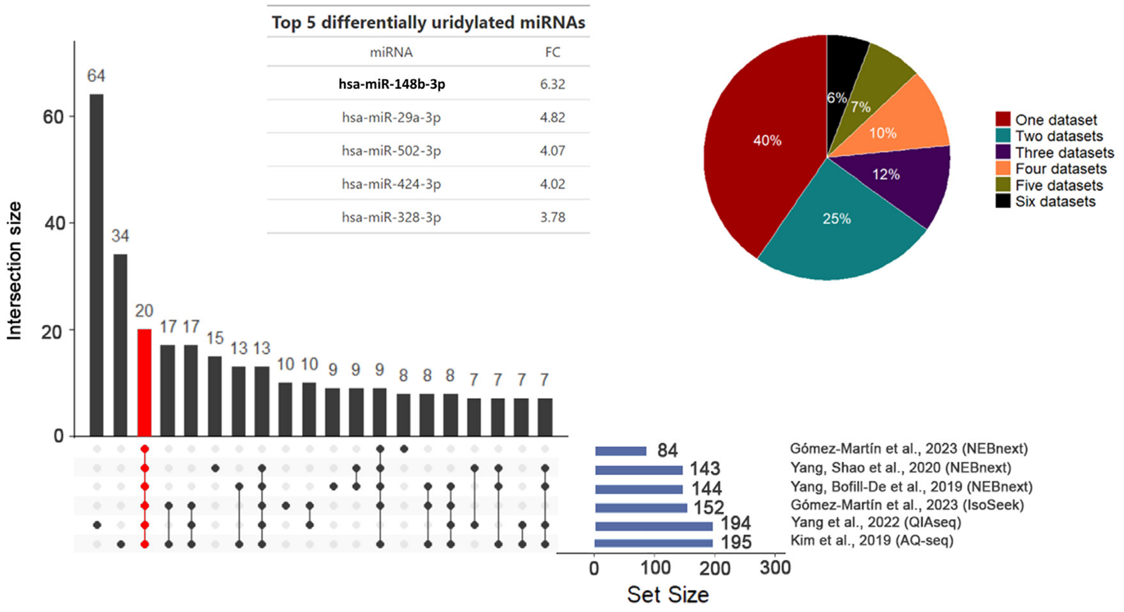

Top 5 differentially uridylated miRNAs

miRNA	FC
**hsa-miR-148b-3p**	6.32
hsa-miR-29a-3p	4.82
hsa-miR-502-3p	4.07
hsa-miR-424-3p	4.02
hsa-miR-328-3p	3.78

Figure 3. Differentially uridylated miRNAs in TUT4/7 DKO HEK293T cells vs. WT from 6 different studies
A total of 20 miRNAs (red-highlighted bar) were found in common between the 6 different studies from 4 different protocols (O/E ratio = 20, *Z* score = 28.5). This represents 6% of the total of differentially uridylated miRNAs (See pie plot, black section). The inset box presents a table with the top 5 (with the highest fold-change [FC]) differentially uridylated miRNAs.

profiling of isomiRs is mandatory to achieve detailed interpretations of miRNA biological functions. Given the growing body of evidence for the functional importance of certain classes of isomiRs,[13–15,19,31–36] we propose that miRNA expression profiling with sequencing is ideally analyzed at the individual isomiR level, rather than aggregating all functional variants

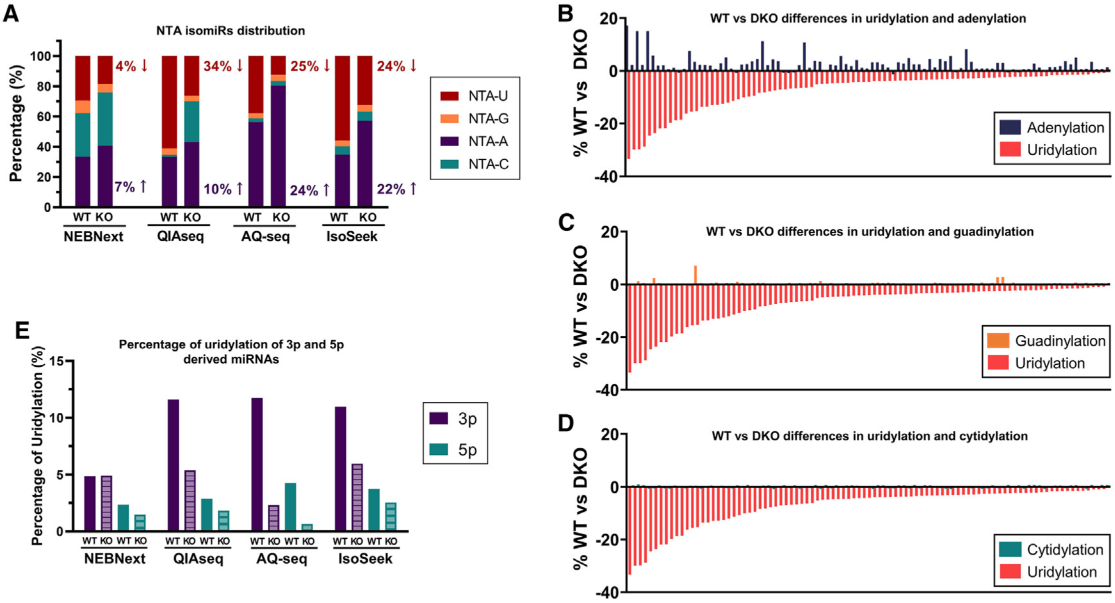

Figure 4. Differences in miRNA uridylation detection between protocols
(A) NTA isomiRs distribution in 4 different protocols (NEBNext, QIAseq, AQ-seq, and IsoSeek) in both WT and TUT4/7 DKO HEK293T cells.
(B–D) Difference of percentage of uridylation and adenylation, guadinylation, and cytidylation, respectively, between the HEK293T parental cells and TUT4/7 DKO cells of the differentially uridylated miRNAs according to IsoSeek.
(E) Percentage of uridylation of miRNAs derived from the 3p arm (purple) or the 5p arm (green) in HEK293T WT and TUT4/7 DKO cells after library preparation using NEBNext, QIAseq, AQ-seq, and IsoSeek.

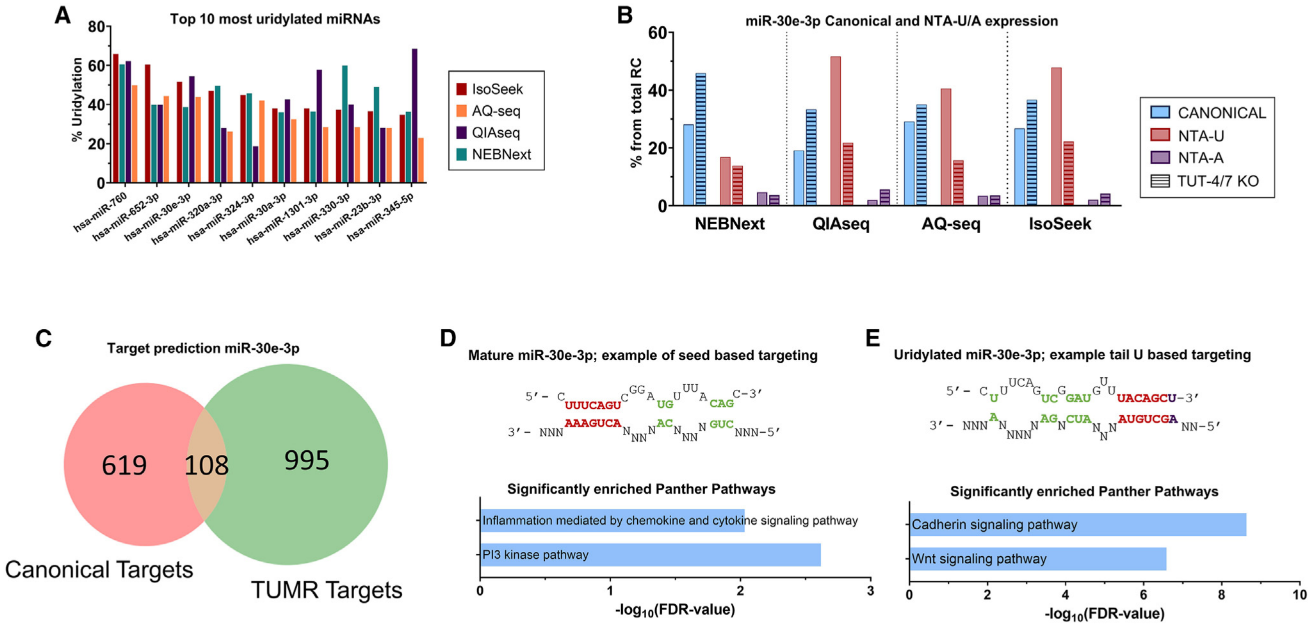

Figure 5. Biased detection of miRNA uridylation may negatively affect target prediction
(A) Top 10 most uridylated miRNAs in HEK293T cells with the highest percentage of uridylation upon use of IsoSeek compared with NEBNext, AQ-seq, and QIAseq.
(B) Percentage of canonical, mono-uridylated, and mono-adenylated miR-30e-3p in HEK293T libraries using NEBNext, QIAseq, AQ-seq and IsoSeek protocols. TUT4/7 DKO cells are represented with striped bars.
(C) Target prediction of mature miR-30e-3p using the canonical seed sequence (red) or the mono-uridylated tail-U based targeting (TUMR, green) and its overlap.
(D and E) Possible 3′ UTR targeting of miR-30e-3p. (D) shows the mature miR-30e-3p sequence and a potential targeting based on its seed sequence. Below are the significantly enriched Panther pathways in the target genes of the canonical isoform. (E) shows an alternative tail-U-based targeting of mono-uridylated miR-30e-3p and the significantly enriched Panther pathways of the TUMR targets.

and non-functional sequences into one miRNA read count number.

Limitations of the study

The main limitation of the study is that, despite conducting a comprehensive comparison of several available miRNA-seq protocols, other less-known or future protocols not included in this study have the potential to outperform those described here. However, by following the same analysis pipeline described here, any other potential protocol should be easily compared with the others.

It is important to note that the choice of the bioinformatics pipeline is crucial, especially for isomiR analysis, and that the analysis with a different bioinformatics pipeline can lead to different results. In this study, we used the widely used and validated miRNA analysis software sRNAbench, which is also optimized for isomiR profiling.

STAR★METHODS

Detailed methods are provided in the online version of this paper and include the following:

- KEY RESOURCES TABLE
- RESOURCE AVAILABILITY
  - Lead contact
  - Materials availability
  - Data and code availability
- EXPERIMENTAL MODEL AND SUBJECT DETAILS
  - HEK293T cells
  - 5N adapters and isomiR spike-ins
- METHOD DETAILS
  - Small RNA library preparation and sequencing
  - RNA isolation
  - Processing of sequencing data and isomiR profiling
  - Prediction of canonical and TUMR miR-30e-3p targets
  - Data visualization
- QUANTIFICATION AND STATISTICAL ANALYSIS
  - Differential expression and statistical analysis of the intersection
  - Pathway enrichment analysis

SUPPLEMENTAL INFORMATION

Supplemental information can be found online at https://doi.org/10.1016/j.crmeth.2023.100480.

ACKNOWLEDGMENTS

This work was supported by Spanish Government (AGL2017-88702-C2-2-R) to M.H., Stitching Cancer Center Amsterdam (CCA2021-9-77) to C.G.-M., and multiple grants awarded to D.M.P. including NWO Perspectief Cancer-ID, TKI-health Holland AQrate, Cancer Center Amsterdam Foundation, and

Stichting MRD in Hodgkin Lymphoma. The authors would like to thank Dr. S. Gu for providing the TUT4/7 DKO HEK293T cells, and the usage of the computational infrastructure of the Computational Epigenomics Lab of the University of Granada.

AUTHOR CONTRIBUTIONS

C.G.-M. performed the analysis together with M.A.J.v.E., E.A.-P., and J.M.M. M.A.J.v.E. performed the experiments. D.M.P. and M.H. co-designed and supervised this study. C.G.-M. and D.M.P. drafted the manuscript. All authors helped to edit the manuscript and final approval.

DECLARATION OF INTERESTS

D.M.P. and M.H. are co-founders of Exbiome BV of which D.M.P. acts as CSO. D.M.P. and M.H. are inventors on a related patent (PCT/EP2015/058614).

Received: August 31, 2022
Revised: March 7, 2023
Accepted: April 21, 2023
Published: May 16, 2023

REFERENCES

1. Gebert, L.F.R., and MacRae, I.J. (2019). Regulation of microRNA function in animals. Nat. Rev. Mol. Cell Biol. *20*, 21–37. https://doi.org/10.1038/s41580-018-0045-7.
2. Drees, E.E.E., and Pegtel, D.M. (2020). Circulating miRNAs as biomarkers in aggressive B cell lymphomas. Trends Cancer *6*, 910–923. https://doi.org/10.1016/j.trecan.2020.06.003.
3. Rupaimoole, R., and Slack, F.J. (2017). MicroRNA therapeutics: towards a new era for the management of cancer and other diseases. Nat. Rev. Drug Discov. *16*, 203–222. https://doi.org/10.1038/nrd.2016.246.
4. Heinicke, F., Zhong, X., Zucknick, M., Breidenbach, J., Sundaram, A.Y.M., T Flåm, S., Leithaug, M., Dalland, M., Farmer, A., Henderson, J.M., et al. (2020). Systematic assessment of commercially available low-input miRNA library preparation kits. RNA Biol. *17*, 75–86. https://doi.org/10.1080/15476286.2019.1667741.
5. Giraldez, M.D., Spengler, R.M., Etheridge, A., Godoy, P.M., Barczak, A.J., Srinivasan, S., De Hoff, P.L., Tanriverdi, K., Courtright, A., Lu, S., et al. (2018). Comprehensive multi-center assessment of small RNA-seq methods for quantitative miRNA profiling. Nat. Biotechnol. *36*, 746–757. https://doi.org/10.1038/nbt.4183.
6. Kim, H., Kim, J., Kim, K., Chang, H., You, K., and Kim, V.N. (2019). Bias-minimized quantification of microRNA reveals widespread alternative processing and 3 end modification. Nucleic Acids Res. *47*, 2630–2640. https://doi.org/10.1093/nar/gky1293.
7. van Eijndhoven, M.A.J., Aparicio-Puerta, E., Gómez-Martín, C., Medina, J.M., Drees, E.E.E., Bradley, E.J., Bosch, L., Scheepbouwer, C., Hackenberg, M., and Pegtel, D.M. (2021). Unbiased and UMI-informed sequencing of cell-free miRNAs at single-nucleotide resolution. Preprint at bioRxiv. https://doi.org/10.1101/2021.05.04.442244.
8. Hu, J.F., Yim, D., Ma, D., Huber, S.M., Davis, N., Bacusmo, J.M., Vermeulen, S., Zhou, J., Begley, T.J., DeMott, M.S., et al. (2021). Quantitative mapping of the cellular small RNA landscape with AQRNA-seq. Nat. Biotechnol. *39*, 978–988. https://doi.org/10.1038/s41587-021-00874-y.
9. Burroughs, A.M., Ando, Y., de Hoon, M.J.L., Tomaru, Y., Nishibu, T., Ukekawa, R., Funakoshi, T., Kurokawa, T., Suzuki, H., Hayashizaki, Y., et al. (2010). A comprehensive survey of 3' animal miRNA modification events and a possible role for 3' adenylation in modulating miRNA targeting effectiveness. Genome Res. *20*, 1398–1410. https://doi.org/10.1101/gr.106054.110.
10. Morin, R.D., O'Connor, M.D., Griffith, M., Kuchenbauer, F., Delaney, A., Prabhu, A.L., Zhao, Y., McDonald, H., Zeng, T., Hirst, M., et al. (2008). Application of massively parallel sequencing to microRNA profiling and discovery in human embryonic stem cells. Genome Res. *18*, 610–621. https://doi.org/10.1101/gr.7179508.
11. Wyman, S.K., Knouf, E.C., Parkin, R.K., Fritz, B.R., Lin, D.W., Dennis, L.M., Krouse, M.A., Webster, P.J., and Tewari, M. (2011). Post-transcriptional generation of miRNA variants by multiple nucleotidyl transferases contributes to miRNA transcriptome complexity. Genome Res. *21*, 1450–1461. https://doi.org/10.1101/gr.118059.110.
12. Valdmanis, P.N., Kim, H.K., Chu, K., Zhang, F., Xu, J., Munding, E.M., Shen, J., and Kay, M.A. (2018). miR-122 removal in the liver activates imprinted microRNAs and enables more effective microRNA-mediated gene repression. Nat. Commun. *9*, 5321. https://doi.org/10.1038/s41467-018-07786-7.
13. Yang, A., Bofill-De Ros, X., Shao, T.J., Jiang, M., Li, K., Villanueva, P., Dai, L., and Gu, S. (2019). 3′ uridylation confers miRNAs with non-canonical target repertoires. Mol. Cell *75*, 511–522.e4. https://doi.org/10.1016/j.molcel.2019.05.014.
14. Qi, Y., Ding, L., Zhang, S., Yao, S., Ong, J., Li, Y., Wu, H., and Du, P. (2022). A plant immune protein enables broad antitumor response by rescuing microRNA deficiency. Cell *185*, 1888–1904.e24. https://doi.org/10.1016/j.cell.2022.04.030.
15. van der Kwast, R.V.C.T., Woudenberg, T., Quax, P.H.A., and Nossent, A.Y. (2020). MicroRNA-411 and its 5′-IsomiR have distinct targets and functions and are differentially regulated in the vasculature under ischemia. Mol. Ther. *28*, 157–170. https://doi.org/10.1016/j.ymthe.2019.10.002.
16. Koppers-Lalic, D., Hackenberg, M., de Menezes, R., Misovic, B., Wachalska, M., Geldof, A., Zini, N., de Reijke, T., Wurdinger, T., Vis, A., et al. (2016). Noninvasive prostate cancer detection by measuring miRNA variants (isomiRs) in urine extracellular vesicles. Oncotarget *7*, 22566–22578. https://doi.org/10.18632/oncotarget.8124.
17. Aparicio-Puerta, E., Gómez-Martín, C., Giannoukakos, S., Medina, J.M., Scheepbouwer, C., García-Moreno, A., Carmona-Saez, P., Fromm, B., Pegtel, M., Keller, A., et al. (2022). sRNAbench and sRNAtoolbox 2022 update: accurate miRNA and sncRNA profiling for model and non-model organisms. Nucleic Acids Res. *50*, W710–W717. https://doi.org/10.1093/nar/gkac363.
18. Kivioja, T., Vähärautio, A., Karlsson, K., Bonke, M., Enge, M., Linnarsson, S., and Taipale, J. (2011). Counting absolute numbers of molecules using unique molecular identifiers. Nat. Methods *9*, 72–74. https://doi.org/10.1038/nmeth.1778.
19. Yang, A., Shao, T.-J., Bofill-De Ros, X., Lian, C., Villanueva, P., Dai, L., and Gu, S. (2020). AGO-bound mature miRNAs are oligouridylated by TUTs and subsequently degraded by DIS3L2. Nat. Commun. *11*, 2765. https://doi.org/10.1038/s41467-020-16533-w.
20. Maguire, S., Lohman, G.J.S., and Guan, S. (2020). A low-bias and sensitive small RNA library preparation method using randomized splint ligation. Nucleic Acids Res. *48*, e80. https://doi.org/10.1093/nar/gkaa480.
21. Yang, A., Bofill-De Ros, X., Stanton, R., Shao, T.-J., Villanueva, P., and Gu, S. (2022). TENT2, TUT4, and TUT7 selectively regulate miRNA sequence and abundance. Nat. Commun. *13*, 5260. https://doi.org/10.1038/s41467-022-32969-8.
22. Khamina, K., Diendorfer, A.B., Skalicky, S., Weigl, M., Pultar, M., Krammer, T.L., Fournier, C.A., Schofield, A.L., Otto, C., Smith, A.T., et al. (2022). A MicroRNA next-generation-sequencing discovery assay (miND) for genome-scale analysis and absolute quantitation of circulating MicroRNA biomarkers. Int. J. Mol. Sci. *23*, 1226. https://doi.org/10.3390/ijms23031226.
23. Ma, S., Saaem, I., and Tian, J. (2012). Error correction in gene synthesis technology. Trends Biotechnol. *30*, 147–154. https://doi.org/10.1016/j.tibtech.2011.10.002.
24. Kume, H., Hino, K., Galipon, J., and Ui-Tei, K. (2014). A-to-I editing in the miRNA seed region regulates target mRNA selection and silencing efficiency. Nucleic Acids Res. *42*, 10050–10060. https://doi.org/10.1093/nar/gku662.

25. Kawahara, Y., Zinshteyn, B., Sethupathy, P., Iizasa, H., Hatzigeorgiou, A.G., and Nishikura, K. (2007). Redirection of silencing targets by adenosine-to-inosine editing of miRNAs. Science *315*, 1137–1140. https://doi.org/10.1126/science.1138050.

26. Qian, Y., Gong, Y., Zou, X., Liu, Y., Chen, Y., Wang, R., Dai, Z., Tasiheng, Y., Lin, X., Wang, X., et al. (2022). Aberrant APOBEC3C expression induces characteristic genomic instability in pancreatic ductal adenocarcinoma. Oncogenesis *11*, 35. https://doi.org/10.1038/s41389-022-00411-9.

27. Medhi, R., Price, J., Furlan, G., Gorges, B., Sapetschnig, A., and Miska, E.A. (2021). RNA uridyl transferases TUT4/7 differentially regulate miRNA variants depending on the cancer cell-type. RNA *28*, 353–370.

28. Gramantieri, L., Pollutri, D., Gagliardi, M., Giovannini, C., Quarta, S., Ferracin, M., Casadei-Gardini, A., Callegari, E., De Carolis, S., Marinelli, S., et al. (2020). MiR-30e-3p influences tumor phenotype through MDM2/TP53 Axis and predicts sorafenib resistance in hepatocellular carcinoma. Cancer Res. *80*, 1720–1734. https://doi.org/10.1158/0008-5472.CAN-19-0472.

29. Lui, W.-O., Pourmand, N., Patterson, B.K., and Fire, A. (2007). Patterns of known and novel small RNAs in human cervical cancer. Cancer Res. *67*, 6031–6043. https://doi.org/10.1158/0008-5472.CAN-06-0561.

30. Kim, H., Kim, J., Yu, S., Lee, Y.-Y., Park, J., Choi, R.J., Yoon, S.-J., Kang, S.-G., and Kim, V.N. (2020). A mechanism for microRNA arm switching regulated by uridylation. Mol. Cell *78*, 1224–1236.e5. https://doi.org/10.1016/j.molcel.2020.04.030.

31. Woods, S., Charlton, S., Cheung, K., Hao, Y., Soul, J., Reynard, L.N., Crowe, N., Swingler, T.E., Skelton, A.J., Piróg, K.A., et al. (2020). microRNA-seq of cartilage reveals an over-abundance of miR-140-3p which contains functional isomiRs. RNA *26*, 1575–1588. https://doi.org/10.1261/rna.075176.

32. Valdmanis, P.N., Gu, S., Chu, K., Jin, L., Zhang, F., Munding, E.M., Zhang, Y., Huang, Y., Kutay, H., Ghoshal, K., et al. (2016). RNA interference–induced hepatotoxicity results from loss of the first synthesized isoform of microRNA-122 in mice. Nat. Med. *22*, 557–562. https://doi.org/10.1038/nm.4079.

33. Pegtel, D.M., Cosmopoulos, K., Thorley-Lawson, D.A., van Eijndhoven, M.A.J., Hopmans, E.S., Lindenberg, J.L., De Gruijl, T.D., Würdinger, T., and Middeldorp, J.M. (2010). Functional delivery of viral miRNAs via exosomes. Proc. Natl. Acad. Sci. USA *107*, 6328–6333. https://doi.org/10.1073/pnas.0914843107.

34. Koppers-Lalic, D., Hackenberg, M., Bijnsdorp, I.V., van Eijndhoven, M.A.J., Sadek, P., Sie, D., Zini, N., Middeldorp, J.M., Ylstra, B., de Menezes, R.X., et al. (2014). Nontemplated nucleotide additions distinguish the small RNA composition in cells from exosomes. Cell Rep. *8*, 1649–1658. https://doi.org/10.1016/j.celrep.2014.08.027.

35. Distefano, R., Tomasello, L., Rampioni Vinciguerra, G.L., Gasparini, P., Xiang, Y., Bagnoli, M., Marceca, G.P., Fadda, P., Laganà, A., Acunzo, M., et al. (2022). Pan-cancer analysis of canonical and modified miRNAs enhances the resolution of the functional miRNAome in cancer. Cancer Res. *82*, 3687–3700. https://doi.org/10.1158/0008-5472.CAN-22-0240.

36. McCall, M.N., Kim, M.-S., Adil, M., Patil, A.H., Lu, Y., Mitchell, C.J., Leal-Rojas, P., Xu, J., Kumar, M., Dawson, V.L., et al. (2017). Toward the human cellular microRNAome. Genome Res. *27*, 1769–1781. https://doi.org/10.1101/gr.222067.117.

37. Huang, X., Lai, S., Qu, F., Li, Z., Fu, X., Li, Q., Zhong, X., Wang, C., and Li, H. (2022). CCL18 promotes breast cancer progression by exosomal miR-760 activation of ARF6/Src/PI3K/Akt pathway. Mol. Ther. Oncolytics *25*, 1–15. https://doi.org/10.1016/j.omto.2022.03.004.

38. Karlsson, M., Zhang, C., Méar, L., Zhong, W., Digre, A., Katona, B., Sjöstedt, E., Butler, L., Odeberg, J., Dusart, P., et al. (2021). A single–cell type transcriptomics map of human tissues. Sci. Adv. *7*, eabh2169. https://doi.org/10.1126/sciadv.abh2169.

39. Androvic, P., Benesova, S., Rohlova, E., Kubista, M., and Valihrach, L. (2022). Small RNA-sequencing for analysis of circulating miRNAs: benchmark study. J. Mol. Diagn. *24*, 386–394. https://doi.org/10.1016/j.jmoldx.2021.12.006.

40. Kozomara, A., Birgaoanu, M., and Griffiths-Jones, S. (2019). miRBase: from microRNA sequences to function. Nucleic Acids Res. *47*, D155–D162. https://doi.org/10.1093/nar/gky1141.

41. Aparicio-Puerta, E., Gómez-Martín, C., Giannoukakos, S., Medina, J.M., Marchal, J.A., and Hackenberg, M. (2020). mirnaQC: a webserver for comparative quality control of miRNA-seq data. Nucleic Acids Res. *48*, W262–W267. https://doi.org/10.1093/NAR/GKAA452.

42. McGeary, S.E., Lin, K.S., Shi, C.Y., Pham, T.M., Bisaria, N., Kelley, G.M., and Bartel, D.P. (2019). The biochemical basis of microRNA targeting efficacy. Science *366*, eaav1741. https://doi.org/10.1126/science.aav1741.

43. Conway, J.R., Lex, A., and Gehlenborg, N. (2017). UpSetR: an R package for the visualization of intersecting sets and their properties. Bioinformatics *33*, 2938–2940. https://doi.org/10.1093/bioinformatics/btx364.

44. Thomas, P.D., Ebert, D., Muruganujan, A., Mushayahama, T., Albou, L.-P., and Mi, H. (2022). PANTHER: making genome-scale phylogenetics accessible to all. Protein Sci. *31*, 8–22. https://doi.org/10.1002/pro.4218.

STAR★METHODS

KEY RESOURCES TABLE

REAGENT or RESOURCE	SOURCE	IDENTIFIER
Deposited data		
miRXplore™ Universal reference pool, sequenced with IsoSeek protocol, this paper	SRA	PRJNA867189
miRXplore™ Universal reference pool, generated by Tewari[5]	SRA	SRP098948
miRXplore™ Universal reference pool, generated by Valihrach[38]	SRA	SRP258941
miRXplore™ Universal reference pool, generated by Guan[20]	SRA	SRP245378
miRXplore™ Universal reference pool, generated by Cao[8]	SRA	SRP228584
miRXplore™ Universal reference pool, generated by Hackl[22]	SRA	SRP348989
HEK293T cells WT and DKO for TUT4-7 enzymes, sequenced with IsoSeek protocol, this paper	SRA	PRJNA867189
HEK293T cells WT and DKO for TUT4-7 enzymes by Kim[6]	SRA	SRP173274
HEK293T cells WT and DKO for TUT4-7 enzymes by Gu[19]	SRA	SRP227370
HEK293T cells WT and DKO for TUT4-7 enzymes by Gu[13]	SRA	SRP165868
HEK293T cells WT and DKO for TUT4-7 enzymes by Gu[21]	SRA	SRP338096

RESOURCE AVAILABILITY

Lead contact
Further information and requests for resources should be directed to and will be fulfilled by the lead contact, D. Michiel Pegtel (d.pegtel@amsterdamumc.nl).

Materials availability
This study did not generate new unique reagents. HEK293T WT and TUT4/7 DKO cells were a kind gift from Dr. S. Gu group.

Data and code availability
- RNA-seq data have been deposited at SRA and are publicly available as of the date of publication. Accession numbers are listed in the key resources table.
- This paper does not report original code.
- Any additional information required to reanalyze the data reported in this paper is available from the lead contact upon request.

EXPERIMENTAL MODEL AND SUBJECT DETAILS

HEK293T cells
HEK293T WT and TUT4/7 DKO cells, a kind gift from Dr. S. Gu, were cultured in DMEM (Gibco), supplemented with 10% FBS (Life Science Group), 100 U/mL penicillin G, 100 μg/mL streptomycin and 1x MEM non-essential amino acids (Thermo Fisher Scientific).

5N adapters and isomiR spike-ins
All adapters and spike-ins were synthesized by and purchased from Eurogentec.

The 5′- and -3′′-adapter sequences are based on the adapters from the NEBNext Multiplex Small RNA Library Prep Kit for Illumina (New England Biolabs) with the addition of 5 random nucleotides (5N) as described by van Eijndhoven et al.[7]

METHOD DETAILS

Small RNA library preparation and sequencing

All small RNA libraries were prepared using the NEBNext Multiplex Small RNA Library Prep Kit for Illumina with fixed NEBNext adapters from the kit or our custom designed 5′- and -3′′-5N-adapters (IsoSeek).[7]

Libraries from the Miltenyi miRXplore Universal pool were prepared from 5fmol to 200 ng total cellular RNA using both NEBNext and Isoseek protocols. Both adapter-sets and the RT primers were 1:2 diluted (5′-adapters 5.63 μM, 3′-adapters 2.5 μM).

RNA isolation

Total RNA from cell lines was isolated using TRIzol reagent (Thermo Fisher Scientific) according to the manufacturers' protocol.

Processing of sequencing data and isomiR profiling

Pre-processing, mapping of adapter trimmed reads and isomiR classification (as stated in Figure S1) were performed using the latest version of sRNAbench[17] command line tool. Default parameters were used for all analysis steps and miRBase[39] was used as miRNA reference.

In the case of Isoseek and NEBNext samples that were sequenced by our group, quality control was carried out using mirnaQC.[40]

IsomiR classification was made using a hierarchical classification schema (see Figure S1). That way all reads assigned to a given miRNA were checked if they belong to one of the following classes.

1. Canonical miRNA: The sequence is exactly the canonical sequence in miRbase.[39]
2. NucVar: The read starts and ends at the same position as the canonical sequence but shows internal sequence variation.
3. NTA: The read has Non-Templated Additions, those meaning extra A, T(U), C or G at the 3′ end.
4. Length variants: The read starts (lv3p) or ends (lv5p) at the same position as the canonical miRNA but it has been extended or shortened at one of its ends.
5. Multiple length variants (mv): The read doesn't start or end as the canonical sequence

Prediction of canonical and TUMR miR-30e-3p targets

Canonical target sites of miR-30e-3p were calculated using TargetScan version 8.0[41] whereas conserved TUMR targets were obtained as previously described,[13] adapting the script provided by the authors. The analysis was performed on a subset of 23 species where miR-30e-3p is fully conserved, which corresponded to 652096 3′-UTRs sequences from the TargetScan database. TUMR targets were searched in these 3′-UTRs sequences using base-pairing with up to 3 G:U wobble pairs. TUMR targets that were not conserved in at least 15 of the 23 studied species were not further considered.

Data visualization

'UpSetR'[42] R package was used in Figure 3 upset plot together with 'ggplot2'.

QUANTIFICATION AND STATISTICAL ANALYSIS

Comparison of the T>C and A>G modifications percentage between the HEK293T WT cells and the 963-miRNA pool.

For the statistical comparison of the percentage distribution of these modifications between the cells and the synthetic pool, a T-test to compare the means was performed, and only q-value lower than 0.05 was showed.

miRXplore Universal reference pool: NEBnext, n = 9 (number of samples); QIAseq, n = 8; AQ-seq, n = 7; IsoSeek, n = 3.

HEK293T cells: NEBnext, n = 15; QIAseq, n = 2; AQ-seq, n = 2; IsoSeek, n = 2.

Differential expression and statistical analysis of the intersection

Differential expression analysis in each study shown in Figure 3 were computed by means of a t-test and the resulting p value was corrected for multiple testing using Benjamini-Hochberg's FDR correction (q-value <0.05 to be considered as differentially uridylated). Only miRNAs with at least 1.5 times more expression in the WT than the DKO are considered as differentially uridylated and therefore shown in Figure 3.

To test if the observed number of commonly TUT dependent uridylated miRNAs is statistically significant, we randomly pick 1000 times the same number of miRNAs from the total of differentially uridylated miRNAs for each of the 6 studies. For each randomization we determine the intersection of the 6 sets. Thus, we obtain the expected value (mean of the randomization runs) and its standard deviation which allows us to calculate the Z score, i.e. the number of standard deviations the observed value is separated from the expectation.

Gómez-Martín et al., 2023, NEBnext, n = 2 (number of samples); Yang, Shao et al. 2020, n = 3; Yang,Bofill-De Ros et al., 2019, n = 3; Gómez-Martín et al. 2023, IsoSeek, n = 2; Yang et al. 2022, n = 4; Kim et al., 2019, n = 3.

Pathway enrichment analysis

Panther Pathway enrichment analysis was performed by means of PantherDB[43] webserver using as a reference all genes expressed by HEK293T cells as according to Human Protein Atlas database (https://proteinatlas.org).[44] An FDR corrected p value of 0.05 was used as cut-off.

Cell Reports Methods, Volume *3*

Supplemental information

Reassessment of miRNA variant (isomiRs) composition by small RNA sequencing

Cristina Gómez-Martín, Ernesto Aparicio-Puerta, Monique A.J. van Eijndhoven, José M. Medina, Michael Hackenberg, and D. Michiel Pegtel

Supplemental Information

Classification	Sequence
Canonical mature miRNA	CAACACCAGUCGAUGGGCUGU
NucVar: miRNA with internal sequence variation	CAACACCAGUCGUUGGGCUGU
NTA: Non Templated Additions	CAACACCAGUCGAUGGGCUGUA
	CAACACCAGUCGAUGGGCUGUU
	CAACACCAGUCGAUGGGCUGUCC
	CAACACCAGUCGAUGGGCUGUG
Length variants	
lv3p: Lenght variants 3′ end	CAACACCAGUCGAUGGGCU--
	CAACACCAGTCGTTGGGCUGUCT
lv5p: Length variants 5′ end	--ACACCAGUCGAUGGGCUGU
	GGCAACACCAGUCGAUGGGCUGU
mv: multiple length variants	GCAACACCAGUCGAUGGGCUG-

Supplementary Figure 1: IsomiR classification, Related to Figure 1.

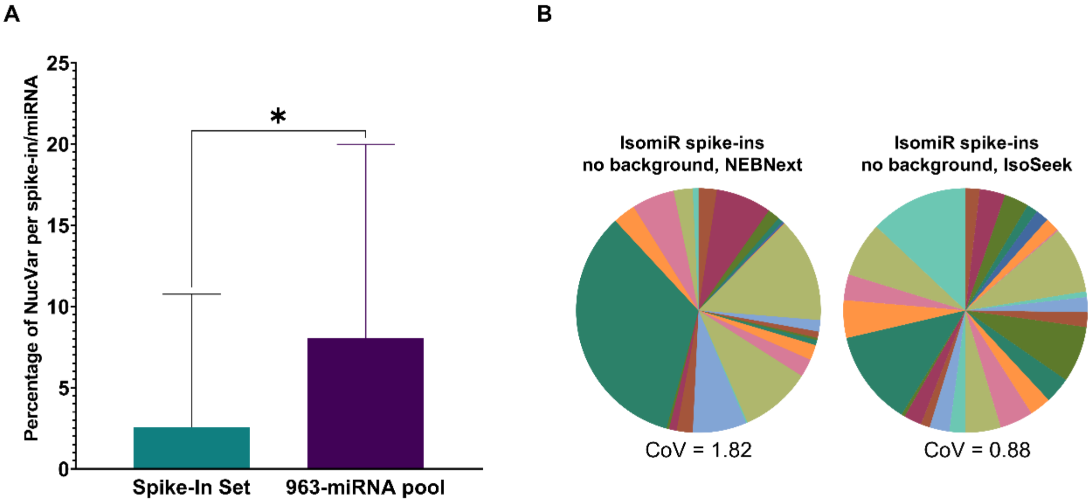

Supplementary Figure 2. Comparison of NucVar isomiRs in the 936-miRNA pool and custom isomiR spike.in set, Related to Figure 2. A) Percentage of NucVar isomiRs per Spike-in/miRNA in the 26 artificial spike-in set (n=3) and the 963-miRNA pool (n=3), both sequenced using Isoseek. The height of the bar represents the mean and the error bar the SD. **B)** Distribution of 26 isomiR spike-ins without biological background. Library preparation is done using NEBNext (left) and IsoSeek (5N) (right).

	TruSeq	CleanTag	NEBNext	AQ-RNA	NEXTflex	QIAseq	IsoSeek	4N-X	4N-G	AQseq
Randomized-end adapters	×	×	×	✓	✓	×	✓	✓	✓	✓
Unique Molecular Identifiers	×	×	×	×	×	12N	5N x 2	×	×	×
Hybridation of RT primer	After both adapters ligation	After both adapters ligation	Between 3' and 5' adapter ligation	Between 3' and 5' adapter ligation	After both adapters ligation	After both adapters ligation	Between 3' and 5' adapter ligation	After both adapters ligation	After both adapters ligation	After both adapters ligation
Other		Optimized structure modifications at the termini of the adapters to avoid adapter dimers					20% PEG at adapter ligation			20% PEG at adapter ligation

Supplementary Table 1. Schematic overview of the different sequencing protocol characteristics, Related to Figure 1.
